# Circumventing AKT-Associated Radioresistance in Oral Cancer by Novel Nanoparticle-Encapsulated Capivasertib

**DOI:** 10.3390/cells9030533

**Published:** 2020-02-25

**Authors:** Liwei Lang, Tiffany Lam, Alex Chen, Caleb Jensen, Leslie Duncan, Feng-Chong Kong, Zoya B. Kurago, Chloe Shay, Yong Teng

**Affiliations:** 1 Department of Oral Biology and Diagnostic Sciences, Dental College of Georgia, Augusta University, Augusta, GA 30912, USA; llang@augusta.edu (L.L.); tlam@augusta.edu (T.L.); jinchen@augusta.edu (A.C.); cajensen@augusta.edu (C.J.); leduncan@augusta.edu (L.D.); zkurago@augusta.edu (Z.B.K.); 2 Georgia Cancer Center, Department of Biochemistry and Molecular Biology, Medical College of Georgia, Augusta University, Augusta, GA 30912, USA; fekong@augusta.edu; 3 Department of Pediatrics, Emory Children’s Center, Emory University, Atlanta, GA 30322, USA; chloe.shay@emory.edu; 4 Department of Medical Laboratory, Imaging and Radiologic Sciences, College of Allied Health, Augusta University, Augusta, GA 30912, USA

**Keywords:** radioresistance, AKT/S6, capivasertib, nanoparticles, OSCC, anticancer

## Abstract

Background: Development of radioresistance in oral squamous cell carcinoma (OSCC) remains a significant problem in cancer treatment, contributing to the lack of improvement in survival trends in recent decades. Effective strategies to overcome radioresistance are necessary to improve the therapeutic outcomes of radiotherapy in OSCC patients. Methods: Cells and xenograft tumors were irradiated using the Small Animal Radiation Research Platform. AKT inhibitor capivasertib (AZD5363) was encapsulated into cathepsin B-responsible nanoparticles (NPs) for tumor-specific delivery. Cell viability was measured by alamarBlue, cell growth was determined by colony formation and 3D culture, and apoptosis was assessed by flow cytometry with the staining of Fluorescein isothiocyanate (FITC) Annexin V and PI. An orthotopic tongue tumor model was used to evaluate the in vivo therapeutic effects. The molecular changes induced by the treatments were assessed by Western blotting and immunohistochemistry. Results: We show that upregulation of AKT signaling is the critical mechanism for radioresistance in OSCC cells, and AKT inactivation by a selective and potent AKT inhibitor capivasertib results in radiosensitivity. Moreover, relative to irradiation (IR) alone, IR combined with the delivery of capivasertib in association with tumor-seeking NPs greatly enhanced tumor cell repression in 3D cell cultures and OSCC tumor shrinkage in an orthotopic mouse model. Conclusions: These data indicate that capivasertib is a potent agent that sensitizes radioresistant OSCC cells to IR and is a promising strategy to overcome failure of radiotherapy in OSCC patients.

## 1. Introduction

Oral squamous cell carcinoma (OSCC) is a major subset of head and neck cancer, representing the 6th most common cancer worldwide [1,2]. Besides surgery, radiotherapy remains a mainstay of treatment for a great majority of patients with OSCC, which improves local tumor control and preserves normal tissues. However, OSCC cells often develop radioresistance, which leads to treatment failure and poor patient prognosis [3,4]. There is a need to better understand the mechanisms of radioresistance in OSCC and to develop effective strategies to counteract it in order to improve the efficacy of radiotherapy.

The serine/threonine protein kinase AKT, a main downstream target of phosphatidylinositol 3-kinase (PI3K), is a central regulator of widely divergent cellular processes, controlling proliferation, differentiation, migration, survival and metabolism [5,6]. Increased AKT activity is common in many types of cancer, including breast, lung, and prostate cancer [6,7]. In human OSCC, persistent AKT activity is also frequent, and it correlates with cancer progression [8]. AKT is constitutively phosphorylated and its kinase activity elevated in many OSCC-derived cell lines [8]. Moreover, the Cancer Genome Atlas (TCGA) and other studies indicate that the most prevalent genetic and epigenetic alterations in OSCC involve the PI3K–AKT–mTOR pathway [9]. Higher levels of p-AKT correlated with worse outcomes in patients with tongue SCC, suggesting that AKT may be a potential target for molecular therapeutics [10]. Most recently, we found that insufficient suppression of the AKT signaling is a major mechanism associated with decrease clinical benefits of Src-targeted treatment in patients with head and neck cancer, and co-inhibition of Src and AKT shows promise for achieving superior anticancer effects in those patients harboring these activations [11].

Aberrant AKT activation is due to molecular alterations of the key regulatory components (e.g., PI3K, PTEN) in its pathway and to the induction of anticancer treatment (e.g., MEK inhibition-induced the compensatory AKT activation). Recent study shows that the upregulation of AKT pathway is a major reason for a poor response to radiation in head and neck cancer [12]. Therefore, specific inhibition of AKT activity may increase the efficacy of radiotherapy. In the present study, we show a link between the activation of AKT/S6 signaling and resistance of human OSCC to irradiation (IR). To address whether AKT activity was directly responsible for radioresistance, we investigated the effect of ATP-competitive AKT inhibitor capivasertib (AZD5363) as a radiosensitizer in OSCC-derived cell lines and preclinical animal models. Moreover, we developed tumor-seeking multifunctional nanoparticles (NPs) to enhance the anti-cancer efficacy of capivasertib, and to reduce its toxicity in normal cells. Our results suggest that radioresistance in OSCC cells can be reversed by capivasertib, providing a potential strategy to improve the therapeutic effects of radiotherapy.

## 2. Materials and Methods

### 2.1. Cell Culture and Standard Assays

SCC25 and Cal27 cells were purchased from the American Type Culture Collection (ATCC; Rockville, MD, USA). HN6 and HN12 cell lines were a gift from Dr. W. Andrew Yeudall and maintained in our lab [13]. All cells were used for experiments before passage 10 and cultured in DMEM containing 10% fetal bovine serum (FBS) at 37 °C in a humidified incubator supplied with 5% CO2. Western blotting and colony formation assays were carried out as previously described [14,15].

### 2.2. Antibodies and Other Reagents

Antibodies that recognize phosphorylated or total proteins, including AKT, p-AKT (S473), S6, p-S6 (240/244), and cleaved poly ADP-ribose polymerase (c-PARP), were purchased from Cell Signaling Technology (Beverly, MA, USA). Antibodies against β-Actin and CD31 were obtained from Abcam (Cambridge, MA, USA). 4′,6-diamidino-2-phenylindole (DAPI) and d-luciferin bioluminescent substrate were purchased from Sigma-Aldrich (St Louis, MO, USA). Capivasertib and LY294002 were purchased from Selleckchem (Houston, TX, USA).

### 2.3. Cell Viability and Apoptosis

Cell viability was measured by alamarBlue Cell Viability Reagent (ThermoFisher Scientific, Waltham, MA, USA) following the indicated treatments. For cultured cells, apoptosis was assessed by flow cytometry using Fluorescein isothiocyanate (FITC) Annexin V Apoptosis Detection Kit (BD Biosciences, San Jose, CA, USA) with Propidium Iodide (PI). In xenograft tumor sections, apoptosis was determined by staining for DNA fragmentation using the DeadEnd^TM^ Fluorometric Terminal deoxynucleotidyl transferase dUTP nick end labeling (TUNEL) System (Promega, Madison, WI, USA). All TUNEL-positive cells in each section in different treatments were counted in twenty randomly selected fields using a fluorescence microscope (Zeiss, Oberkochen, Germany).

### 2.4. Establishment of Radioresistant HN6 (HN6R) Colonies

HN6 cells were irradiated at a dose of 2 Gy. After that, the cells were cultured, split 1:3, allowed to achieve 80% confluence, and then exposed to another cycle of 2 Gy. This process was repeated for a cumulative total of 32 Gy. The resulting cell population was plated at a low density on soft agar, and single cell colonies were picked and expanded in culture. These single colonies were screened by cell viability and colony formation assays after IR at different doses, and the most radioresistant colonies (HN6R#1, HN6R#2) were selected for further study.

### 2.5. Synthesis and Characterization of the Multifunctional Polymeric NPs for Capivasertib Delivery

Linear-dendritic mPEG5000-BMA4 containing four branches of amine groups, the cathepsin B (CTSB)-sensitive polymeric drug carrier, was synthesized as described previously [11,16]. To prepare NP-encapsulated capivasertib (Nano-cap), 1 mg capivasertib and 10 mg amphiphilic polymer were first dissolved in anhydrous chloroform/methanol (1/1) in a 10 mL round- bottom flask. The solvent mixture was evaporated under vacuum to form a thin film. Phosphate-buffered saline (PBS) buffer (1 mL) was added to re-hydrate the thin film, followed by 30 min of sonication. Free drugs not associated with the NPs were removed by running the NP solutions through centrifugal filter devices (MWCO: 3.5 kDa, Microcon). The drug-loaded formulation on the filters were recovered with PBS. The amount of capivasertib loaded in the NPs was analyzed by High-Performance Liquid Chromatography (HPLC) (Agilent 1200 LC, Santa Clara, CA, USA). The drug loading was calculated according to the calibration curve between the HPLC area values and concentrations of drug standard. The size and size distribution of Nano-cap were measured by dynamic light scattering (DLS) instrument three times with an acquisition time of 30 s at room temperature. The drug release profile of Nano-cap was measured in the solution with or without CTSB.

### 2.6. Three-Dimensional (3D) Cell Cultures

Briefly, 2×105 HN12 cells were seeded into 48-well SeedEZ (Lena Bioscience, Atlanta, GA, USA), a completely inert and transparent glass fiber scaffold, supplied with complete medium. After 4 days of culture, cells growing in SeedEZ were exposed to 5 µM Nano-cap and 4 Gy IR alone or in combination. On day 7 after treatment, cell viability in SeedEZ scaffold was measured by alamarBlue at 545/590 nm ex/em, followed by DAPI staining and imaging.

### 2.7. Animal Studies

Six-week-old NOD.Cg-*Prkdc^scid^ Il2rg^tm1Wjl^*/SzJ (NSG) mice were purchased from the Jackson Laboratory (Bar Harbor, ME). All animal experiments were approved by the Institutional Animal Care and Use Committee (IACUC) of Augusta University. To generate an orthotopic xenograft tongue tumor model, 5×104 luciferase-containing HN12 cells were suspended in 50 μL of PBS/Matrigel (3:1) and injected into the anterior portion of the tongue of NSG mice. On day 10 after injection, mice were randomized to receive different treatments: vehicle (PBS), Nano-cap, IR or Nano-cap plus IR. For IR, tumor-bearing mice were anesthetized with isoflurane, the tongue with tumor was located by CT scan and a total dose of 12 Gy IR was delivered using 4Gy fractions per day for 3 days in the Small Animal Radiation Research Platform (SARRP, Xstrahl, GA, USA) [17]. For drug treatment, four doses of 10 mg/mL Nano-cap were administered intravenously every other day. Mice were intraperitoneally injected with D-luciferin bioluminescent substrate, followed by determination of bioluminescence on day 10 (start of treatment) and day 18 (end of experiment) using a Xenogen IVIS-200 In Vivo Imaging System (PerkinElmer, Waltham, MA, USA). Afterward, the mice were sacrificed and the xenografts and major organs (including the heart, intestine, kidney, liver, lung, and spleen) were excised for histopathological analysis with hematoxylin and eosin (H&E) staining and Western blotting. For all of the animal studies, we ensured randomization and blinded conduct of the experiments. For all imaging analyses, an observer who was blind to the experimental groups performed the quantitation. No samples or animals were excluded from the analysis.

### 2.8. Immunohistochemistry (IHC)

Sections of paraffin-embedded mouse tongue tumors derived from HN12 cells were deparaffinized and rehydrated by standard pathology laboratory methods, followed by heat-induced antigen retrieval and IHC with antibodies against p-AKT (1:500), p-S6 (1:500) and CD31 (1:800). Negative controls included non-specific polyclonal rabbit antibody at 2 μg/mL (Abcam, Cambidge, MA, USA). The sections were developed with the diaminobenzidine tetrahydrochloride (DAB) substrate kit (Vector Laboratories, Burlingame, CA, USA) according to the manufacturer’s recommendations and counterstained with hematoxylin. At least five areas for each sample were imaged at random by a CCD camera (Zeiss, Oberkochen, Germany), and IHC staining of the indicated antibody was quantified using Image pro-Plus6.0 software (Media Cybernetics, Silver Springs, MD, USA) and presented as integrated optical density (IOD).

### 2.9. Statistical Analysis

Student’s *t* test was used for comparison of two groups, and analysis of variance (ANOVA) with post-hoc Tukey’s test was used for comparison of multiple groups. Data are expressed as the mean ± SEM. The differences of *p* < 0.05 were considered statistically significant.

## 3. Results

### 3.1. Increased AKT Activation Is Associated with OSCC Radioresistance

**Figure 1:**
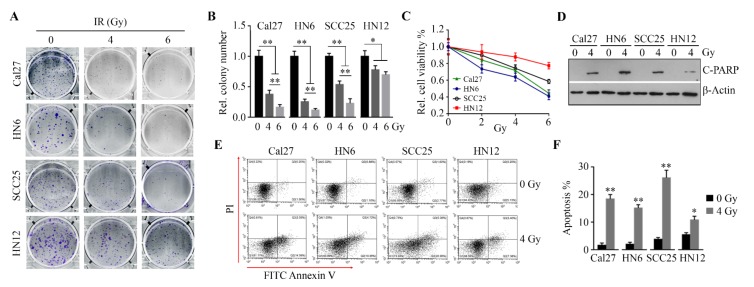
Oral squamous cell carcinoma (OSCC) cells exhibit differential responses to irradiation (IR). (**A, B**) The effects of IR on the ability of OSCC cell lines to form colonies were determined on Day 14 after IR. The representative results and quantitative data from three independent experiments are shown in (**A**) and (**B**), respectively. (**C**) The effects of IR on OSCC cell viability were determined on Day 3 after IR. (**D**) The effect of IR on poly ADP-ribose polymerase (PARP) cleavage were determined in OSCC cell lines on Day 3 after IR. (**E, F**) The effects of IR on apoptosis were determined in OSCC cell lines using Fluorescein isothiocyanate (FITC) Annexin V Apoptosis Detection Kit with PI on Day 3 after IR. A representative result and quantitative data from three independent experiments are shown in (**E**) and (**F**), respectively. * *p* < 0.05; ** *p* < 0.01.

To determine the radiosensitivity of OSCC cells, four OSCC cell lines (Cal27, HN6, SCC25 and HN12) were irradiated using a range of doses. Colony formation and viability assays showed that IR abolished cell clonogenicity (Figure 1A,B), as well as reduced cell survival (Figure 1C). The analysis of apoptosis by Western blotting with antibody against c-PARP (Figure 1D) or by flow cytometry upon Annexin V and PI staining (Figure 1E,F), revealed that IR induced apoptosis in all four cell lines. However, HN12 cells were less sensitive to IR than the other three cell lines (Figure 1A–F). Moreover, HN12 cells did not exhibit a dose-dependent response to IR on colony formation, as evidenced by no significant changes in cell colony number when exposed to IR at different dose-rates (4 Gy vs. 6 Gy) (Figure 1A,B). These findings indicate that HN12 cells are more resistant to IR than the other three OSCC cell lines.

**Figure 2:**
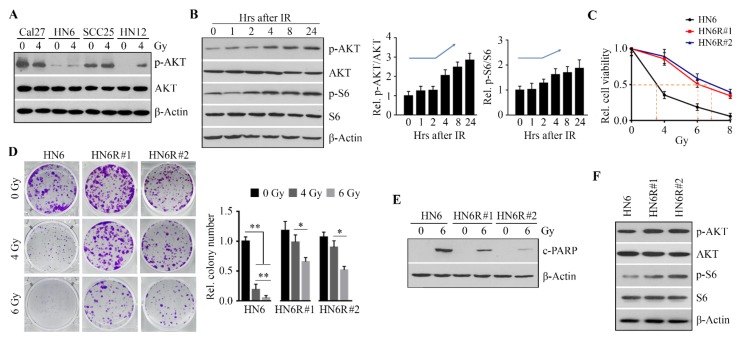
The upregulation of AKT/S6 signaling confers OSCC cells radioresistance. (**A**) AKT activity in OSCC cell lines one day after IR. (**B**) The levels of p-AKT and p-S6 in HN12 cells within 24 h following IR. A representative Western blotting and quantitative data from three independent experiments are shown in the left and right panels, respectively. (**C**) The effects of IR on viability of radioresistant HN6R#1 and HN6R#2 cells and radiosensitive parental HN6 cells were determined by alamarBlue on Day 5 after IR. (**D**) The effects of IR on the ability of radioresistant and radiosensitive HN6 cells to form colonies were measured on Day 14 after IR. A representative result and quantitative data from three independent experiments are shown in the left and right panels, respectively. (**E**) The effects of IR on PARP cleavage in radioresistant and radiosensitive HN6 cells were determined on Day 3 after IR. (**F**) p-AKT and p-S6 levels in radioresistant and radiosensitive HN6 cells on Day 1 after IR. * *p* < 0.05; ** *p* < 0.01.

We next examined the status of p-AKT in OSCC cell lines before and after IR. Compared with the other three radiosensitive cell lines, increased p-AKT was only observed in HN12 cells exposed to IR (Figure 2A), suggesting that AKT activation may correlate with OSCC radioresistance. Moreover, the phosphorylation levels of AKT were increased at 4 h in irradiated HN12 cells, and the high levels of p-AKT lasted at least 20 h after IR (Figure 2B). The phosphorylation levels of ribosomal protein S6 (S6), a major downstream target of AKT, were also increased in HN12 cells following IR, which was similar to the changes in p-AKT (Figure 2B). Compared with HN12 cells, HN6 cells were more sensitive to IR (Figure 1). To validate the results obtained with HN12 cells, we used HN6 cells to generate radioresistant HN6R by exposing HN6 cells to a cumulative total of 32 Gy. HN6R#1 [the half maximal inhibitory concentration (IC50) = 6.1 Gy] and HN6R#2 (IC50 = 6.9 Gy) were the most radioresistant colonies with tolerance to IR at 4 Gy, as evidenced by the lack of significant decrease in cell viability at this dose compared with untreated HN6R cells (Figure 2C). Although IR at 6 Gy significantly decreased cell viability and colony formation, as well as increased apoptosis in HN6R#1 and HN6R#2, the sensitivity of these two colonies to IR was significantly less than that of the parental cells HN6 (Figure 2C–E). Most importantly, both p-AKT and p-S6 levels were higher in two HN6R colonies than those in parental cells (Figure 2F), which was in line with the observations in HN12 cells with or without IR. Collectively, these data indicate that the activation of the AKT/S6 signaling axis is associated with differential radiosensitivity in OSCC cells.

### 3.2. Inactivation of the AKT Signaling by Capivasertib Improves the Anticancer Activity of IR in OSCC Cells

**Figure 3:**
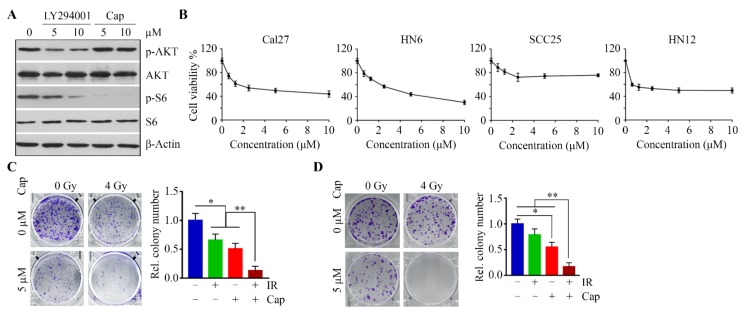
Suppressing AKT/S6 signaling sensitizes OSCC cells to IR. (**A**) The effects of LY294002 and capivasertib (Cap) on S6 phosphorylation in HN12 cells on Day 1 after treatment. (**B**) The effects of capivasertib on viability of OSCC cell lines were determined on Day 3 after treatment. (**C**) The effects of combined capivasertib and IR treatment on the ability of HN12 cell to form colonies were determined on Day 14 after treatment. A representative result and quantitative data from three independent experiments are shown in the left and right panels, respectively. (**D**) The effect of combined capivasertib and IR treatment on the ability of HN6R cells to form colonies was determined on Day 14 after treatment. A representative result and quantitative data from three independent experiments are shown in the left and right panels, respectively. * *p* < 0.05; ** *p* < 0.01.

LY294002 can reduce AKT phosphorylation through inhibition of its upstream enzyme PI3K, while capivasertib is an ATP-competitive AKT inhibitor with the great potential to prevent the phosphorylation of AKT substrates [11]. We treated HN12 cells with these two inhibitors and compared the inhibitory effect on AKT signaling. As shown in Figure 3A, capivasertib at 5 µM completely inhibited the phosphorylation of S6 in HN12 cells, which was more potent than LY294002 at the same dose (Figure 3A). We then determined the IC50 of capivasertib among the OSCC cell lines. The IC50 of capivasertib in three out of four cell lines (Cal27, HN6, and HN12) was approximately 5 µM (Figure 3B). At this dose, capivasertib enhanced radiosensitivity of HN12 (Figure 3C) and re-sensitized HN6R cells to IR (Figure 3D).

**Figure 4:**
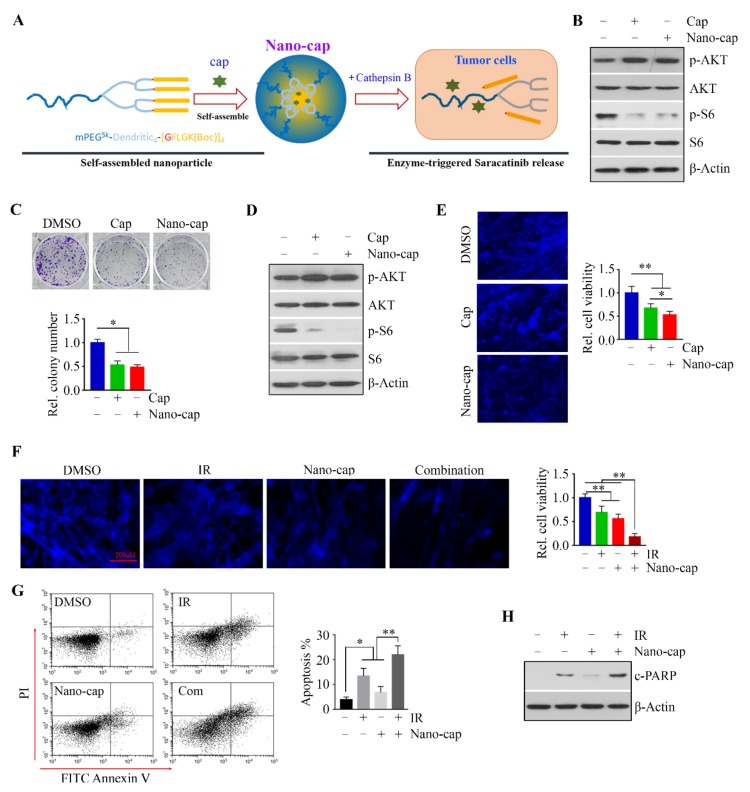
The addition of Nano-cap to IR greatly strengthens the cytotoxic effect on OSCC cells in a 3D SeedEZ scaffold. (**A**) Schematic representation of the self-assemble Nano-cap and its disassembly upon CTSB digestion. (**B**,**D**) The effects of Cap and Nano-cap on the p-AKT and p-S6 levels in HN12 cells were determined on Day 1 after treatment. Cells were seeded in a 2D culture dish (**B**) or SeedEZ scaffold (**D**). (**C**) The effects of Cap and Nano-cap on HN12 cell colony forming ability were determined on Day 14 after treatment. A representative result and quantitative data from three independent experiments are shown in the upper and lower panels, respectively. (**E**) The effects of Cap and Nano-cap on HN12 cell viability in 3D SeedEZ scaffold were determined on Day 7 after treatment. Representative images of 4′,6-diamidino-2-phenylindole (DAPI) staining and quantitative data of cell viability measured by alamarBlue are shown in the left and right panels, respectively (**F**) The effects of Nano-cap and IR alone or in combination on HN12 cell viability were determined on Day 7 after treatment. Representative images of DAPI staining and quantitative data of cell viability measured by alamarBlue are shown in the left and right panels, respectively. (**G**) The effects of Nano-cap and IR alone or in combination on HN12 cell apoptosis was determined on Day 3 after treatment. (**H**) The effects of Nano-cap and IR alone or in combination on PARP cleavage in HN12 cells were determined on Day 3 after treatment. * *p* < 0.05; ** *p* < 0.01.

In order to enhance capivasertib delivery to oral tumor cells, capivasertib was dissolved and encapsulated into nano-matrix with the enzyme-cleavable peptidic linker GFLG, which is sensitive to the lysosomal enzyme CTSB for drug release (Figure 4A). DLS analysis in our previous study has shown that Nano-cap displayed narrow, monomodal particle size distributions, with a z-average size of approximately 58.3 nm and polydispersity index (PDI) of approximately 0.16 [11]. Moreover, more than 80% capivasertib can be released from Nano-cap within 48 h in the presence of CTSB at pH 5.4, indicating a CTSB-responsive drug release profile [11]. We then compared the effects of 5 µM free capivasertib and Nano-cap in HN12 cells over the 24 h treatment period. Both treatments increased p-AKT, but markedly inhibited phosphorylation of S6 (Figure 4B). Because these two treatments resulted in similar reductions in S6 phosphorylation and colony formation (Figure 4B,C), we compared their efficacy in HN12 cells in a more physiologically relevant setting. We turned to 3D cultures using the SeedEZ scaffold, in which p-S6 levels and cell viability were suppressed more efficiently by Nano-cap than by free drug (Figure 4D,E). These data support a superior inhibitory effect of Nano-cap on OSCC cell survival in 3D culture system.

To examine the combined effects of Nano-cap and IR, we cultured HN12 cells in the SeedEZ scaffold, followed by treatment with Nano-cap and IR alone or in combination. The addition of Nano-cap to IR dramatically decreased cell viability in 3D culture compared with single-arm treatment alone (Figure 4F,G), which was in line with the data obtained in 2D cultures (Figure 3). To determine apoptosis, cells with different treatment were trypsinized from SeedEZ and stained with Annexin V and PI solution followed by flow cytometry analysis. As shown in Figure 4H, the expression of c-PARP as a measure of apoptosis was markedly increased when cells were exposed to Nano-cap + IR relative to either treatment alone. These observations support the notion that the addition of Nano-cap to IR greatly increases OSCC cell death.

### 3.3. Nano-Cap Potentiates the Efficacy of IR in the Treatment of Oral Tumors in the Orthotopic Xenograft Mice Model

**Figure 5:**
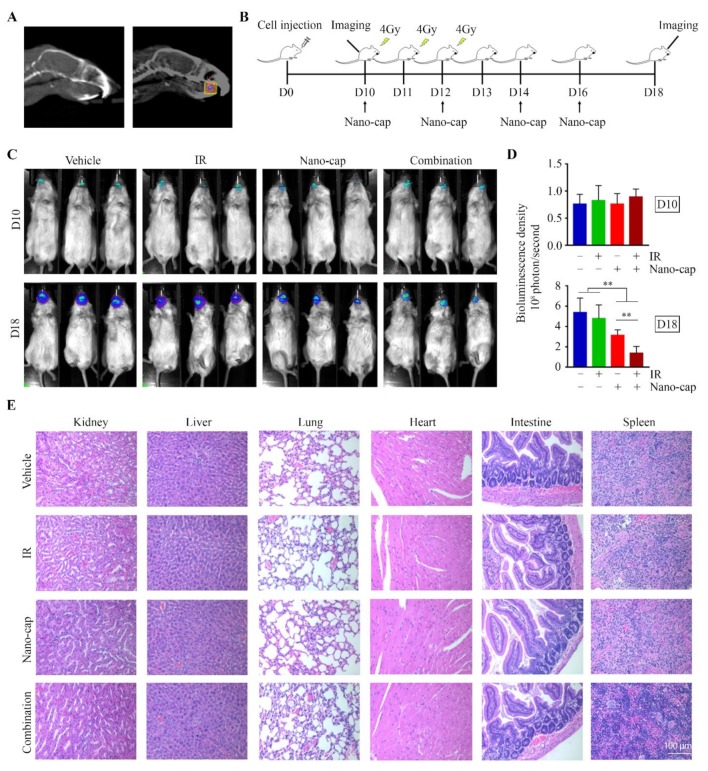
Combined Nano-cap and IR treatment greatly enhances the negative effect on tumor growth relative to monotherapy in HN12-derived orthotopic xenograft mouse model. (**A**) CT images of the mouse head. Right panel, the square area inside the box indicates the location of the tumor xenograft in the tongue, which is the IR target. (**B**) The timeline of experimental procedures in vivo. HN12-derived tumor-bearing mice (*n* = 10/group) received IR and/or Nano-cap on day 10 (D10) after cell inoculation into tongue and were sacrificed on D18. (**C**) Representative bioluminescence images from the Xenogen IVIS-200 In Vivo imaging system showing the size of tongue tumors in mice at the beginning (D10) and the end (D18) of the indicated treatments. (**D**) Quantitative data (*n* = 10) of bioluminescence density of tongue tumors on D10 and D18. (E) Histology of major organs at the endpoint of each treatment. ** *p* < 0.01.

Based upon the strong in vitro 3D data, we next evaluated the therapeutic efficacy of Nano-cap in combination with IR in vivo. NSG mice were inoculated with HN12 cells to establish tongue xenografts. Mice with established xenografts (day 10) were randomized into four groups to receive different treatments: vehicle, Nano-cap, local IR to the tongue, or Nano-cap combined with local IR (Figure 5A,B). Fractionated IR alone (for total 12 Gy) did not lead to significant shrinkage of tumor xenografts within 8 days post-exposure, as measured by bioluminescence (Figure 5C,D). However, there was a reduction in tumor growth in mice that received a single Nano-cap treatment (Figure 5C,D), which was more pronounced in response to the combined IR and Nano-cap treatment (Figure 5C,D). Analysis of the major organs, including spleen, heart, intestines, kidney, liver and lung, revealed no morphologic changes in mice receiving either single or combination treatments (Figure 5E), indicating that Nano-cap does not produce detectable systemic toxicity.

**Figure 6:**
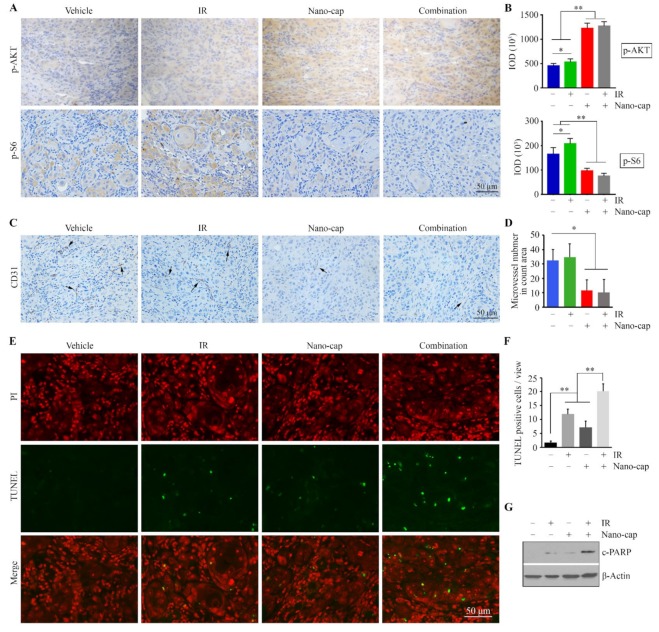
Nano-cap improves IR efficacy through suppressing AKT/S6 signaling and neovascularization in HN12-derived orthotopic xenograft mice. (**A**,**B**) p-AKT and p-S6 levels in tumor xenografts from mice receiving different treatments were determined by immunohistochemistry (IHC). Representative IHC images and quantification of IHC staining using Image pro-Plus6.0 are shown in (**A**,**B**), respectively. (**C**) CD31-postitive microvessels (arrows) in tumor xenografts from mice receiving different treatments were determined by IHC. (**D**) Quantification of the CD31-positive microvessels. (**E**,**F**) Apoptosis in tumor xenografts from mice receiving different treatments was determined by the Terminal deoxynucleotidyl transferase dUTP nick end labeling (TUNEL) assay. Representative TUNEL-stained xenograft sections are shown in (**E**) and quantification of TUNEL-positive tumor cells is shown in (**F**). (**G**) The effects of indicated treatments on PARP cleavage in the orthotopic xenograft model. * *p* < 0.05; ** *p* < 0.01.

To address the mechanism underlying the action of combined treatment, tumor xenograft sections were immunostained with the antibodies against p-AKT and p-S6. In line with the in vitro data from 2D and 3D cultures, IR increased both AKT and S6 phosphorylation levels in tumor cells, and the addition of Nano-cap significantly attenuated IR-induced upregulation of AKT/S6 signaling (Figure 6A,B). In addition, we evaluated the tumor vasculature using immunohistochemistry for CD31 as a potential cause of tumor shrinkage. We found a slight increase in the number of CD31-positive microvessels in tumor xenografts exposed to IR only as compared with vehicle-treated group (Figure 6C,D). However, there was a significant reduction in the number of CD31-positive microvessels in the tumor xenografts treated with Nano-cap or Nano-cap + IR relative to treatment with vehicle or IR only (Figure 6C,D). These observations are in keeping with a previous study [18], indicating that capivasertib contributes to marked reduction in tumor vascularity in vivo, which may contribute to the observed inhibitory effect on tumor growth. Moreover, we found that Nano-cap + IR induced more tumor cell apoptosis as measured by TUNEL assays (Figure 6E–F). Similarly, Nano-cap combined with IR enhanced c-PARP levels in xenograft tumor cells as compared to either single treatment (Figure 6G). To validate the tumor-specific delivery of Nano-cap, we further examined the level changes in p-S6 among major organs removed from mice treated with vehicle or Nano-cap by Western blotting. This analysis revealed that Nano-cap leads to reduced p-S6 levels in tumor xenografts rather than these major mouse organs (Supplementary Information). Taken together, these data suggest that Nano-cap has the great potential to strongly enhance the therapeutic efficacy of IR in OSCC, at least in part through inactivating AKT/S6 signaling.

## 4. Discussion

OSCC carries a poor prognosis with minimal improvement in survival trends in decades. Fractionated radiotherapy with ionizing radiation is a mainstay of OSCC patient treatment, but development of radioresistance frequently results in treatment failures. In this study, we have found that upregulation of the AKT/S6 activation is an important mechanism by which OSCC cells can develop radioresistance. To better demonstrate the importance of AKT/S6 signaling in IR-induced OSCC resistance, we generated radioresistant OSCC cells, as well as the orthotopic mice of OSCC receiving IR to the local tumors, which are particularly relevant for modeling radioresistant in OSCC. Capivasertib, the selective and potent AKT inhibitor, can reduce radioresistance and improve the efficacy of radiotherapy in 3D cell cultures and the preclinical animal model, with a tumor-seeking nano-based drug delivery system. These findings suggest that capivesartib-mediated AKT/S6 inhibition may be useful for the treatment of patients with radioresistant OSCC.

AKT acts as a pivotal point of converging signaling pathways involved in cell proliferation, differentiation, migration, survival and metabolism [5,6]. Excessive activation of AKT in OSCC has been implicated as a key step paralleling the severity and progression stage of OSCC [8]. In this regard, targeting AKT is a potentially useful strategy that may improve patient outcomes. PI3K lies upstream of AKT, and PI3K inhibitors have been noted to contribute to AKT inhibition. Although PI3K inhibitors are currently in late-stage clinical trials, the development of optimal combination regimens is likely to be a challenge for graduation into clinical practice [19]. In the present work, capivasertib was a more potent inhibitor of AKT/S6 signaling than the PI3K inhibitor LY294002 at the same dose, suggesting that directly targeting AKT by capivasertib leads to a superior effect on S6 inactivation in OSCC cells. It was noted that further molecular effects are related to AKT/S6 activation in cancer cells. In particular, AKT/S6 signaling is associated with the functions of Survivin, which is an anti-apoptotic protein highly expressed in OSCC and explored as a therapeutic anticancer target [20]. Increased apoptosis is evident in capivasertib-treated OSCC cells, and thus, further exploration whether apoptosis induced by this drug is due to inactivation of the AKT/S6/Survivin signaling is warranted. 

Although recent attempts to identify specific inhibitors with acceptable pharmacological properties have been pursued, clinically useful AKT inhibitors are currently not available. The failure in using AKT inhibitors as a single agent for cancer treatment prompted us to test whether they can be applied in combination therapy. In fact, AKT pathway alterations have been implicated in radioresistance in many types of cancer, including head and neck cancer, prostate cancer, glioblastoma, and acute myeloid leukemia [4,9,21,22,23,24,25,26]. We show that the upregulation of the AKT/S6 signaling axis is the major pathway conferring OSCC cells radioresistance, and inhibiting it significantly reverses the radioresistance in OSCC cells. Thus, we determined the effects of AKT inhibitor capivasertib in combination with IR in OSCC, which signifies the strong therapeutic potential of this combined modality treatment in OSCC.

Malignant progression of most benign tumors is typically associated with a transition from a quiescent to a proliferative vasculature in order to satisfy their demand for oxygen and nutrients and accomplish other metabolic functions [27,28]. Capivasertib given following IR to FaDu (the cancer cell line derived from hypopharyngeal squamous cell carcinoma) and PE/CA PJ34 (the cancer cell line derived from basaloid squamous cell carcinoma) tumors was associated with marked reductions in tumor vascular density, which appeared to be secondary to the effects on the irradiated tumor microenvironment as capivasertib had no noticeable impact on tumor control when administered prior to IR [18]. These findings might be arguable due to some limitations, such as the application of animal models and the uptake rate of drug in tumor cells. Here, we show that capivasertib not only represses neovascularization but also inhibits tumor survival in the orthotopic mice of tongue tumors. Together, the observed effects of capivasertib directly on the tumor cells and the tumor vasculature likely contribute to the increased OSCC sensitivity to radiotherapy in vivo, presenting an attractive candidate for further investigation of its use as an adjuvant in cancer therapy.

Tumor-seeking NPs can be achieved by modulating the tumor microenvironment to disrupt some barriers for NP delivery [16]. CTSB belongs to a family of lysosomal cysteine proteases, whose expression levels are significantly increased in serum of patients with colorectal cancer compared with matched normal colon subjects [29], suggesting that CTSB may represent a potential target for NP-based drugs in the treatment of colorectal cancer. Interestingly, profound CTSB upregulation was also found in head and neck cancer tissues compared with paired adjacent normal tissues [30]. Based on this finding, we developed CTSB-degradable NPs for capivasertib delivery, which have the great potential for application in clinical radiotherapy by taking advantage of enhanced drug efficacy in OSCC cells as well as reduced systematic toxicity.

OSCC is frequently associated with poor prognosis due to the high risk of cervical lymph node metastasis even when the tumors are small (T1 and T2). We have generated the orthotopic mouse model for tongue tumors by injection of OSCC cells to NSG mice tongue [11,14]. However, increased tumor size in the tongue could stop mice from eating and tumor-bearing mice often reach a moribund state before cervical or distant metastasis is established from tongue primary tumors. The novel treatment approach present in this study may also affect OSCC metastasis but before testing this, resection of the primary tumor or finding a possible way to expend survival time of tumor-bearing mice is needed to allow the development of metastases.

## 5. Conclusions

In summary, inhibition of the AKT/S6 pathway provides optimism in reversing radioresistance in OSCC, laying the foundation for further clinical investigation into capivasertib as a potential clinical radiosensitizer.

## Supplementary Materials

Supplementary Materials: The following are available online at https://www.mdpi.com/2073-4409/9/3/533/s1, Figure S1: Representative Western blotting results and quantitative data (n = 6) showing the changes in p-S6 levels in tumor xenografts (A) and major mouse organs (B), respectively. 1 and 2 represent the testing samples from two different mice in the same treatment group. ** *p* < 0.01.
