# Supplementary material for: Circumventing AKT-Associated Radioresistance in Oral Cancer by Novel Nanoparticle-Encapsulated Capivasertib
Source: Cells. 2020 Feb 25;9(3):533. doi: 10.3390/cells9030533 (PMC7140405; doi:10.3390/cells9030533)
Supplement: Supplementary file 1 [file cells-09-00533-s001.pdf]

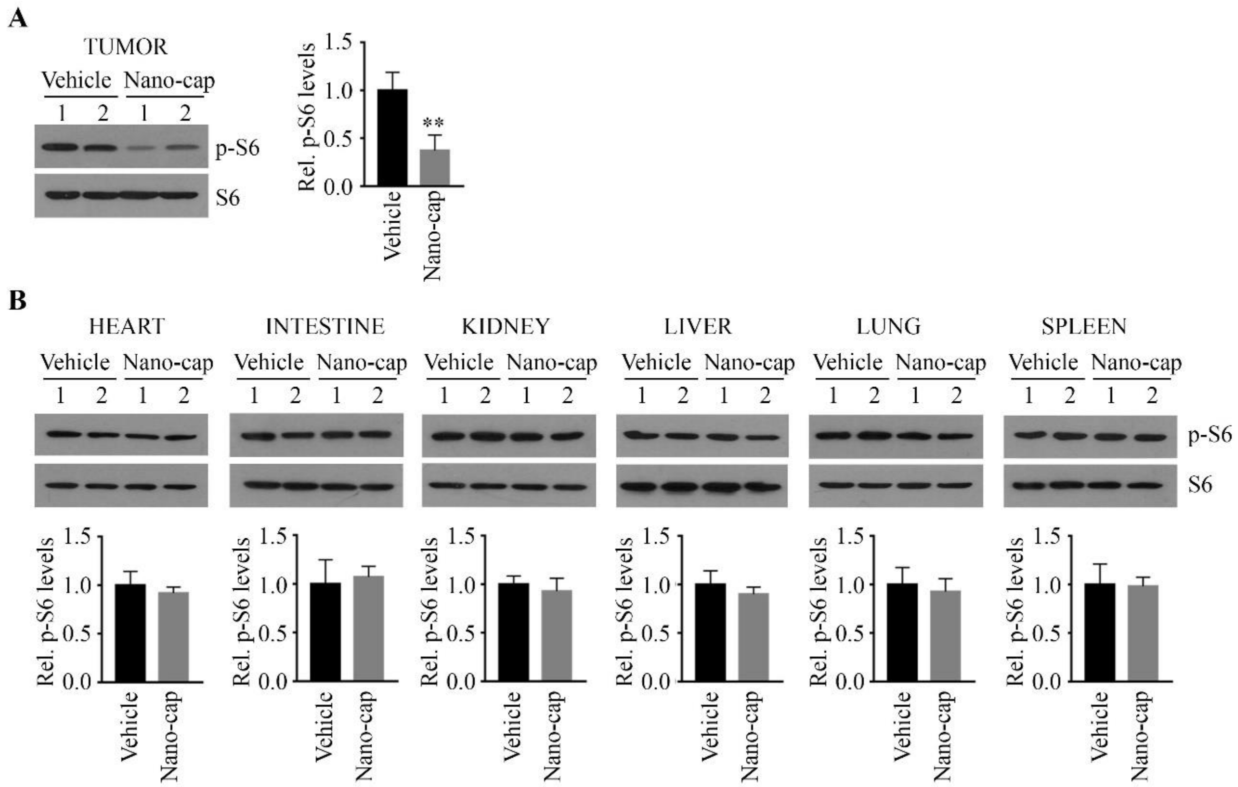

Supplementary Figure 1: Representative Western blotting results and quantitative data (n=6) showing the changes in p-S6 levels in tumor xenografts (**A**) and major mouse organs (**B**), respectively. 1 and 2 represent the testing samples from two different mice in the same treatment group. **$p < 0.01$.
